# Maladaptive Personality Functioning and Psychopathological Symptoms in Problematic Video Game Players: A Person-Centered Approach

**DOI:** 10.3389/fpsyg.2019.02559

**Published:** 2019-11-19

**Authors:** Alessandro Musetti, Tiziana Mancini, Paola Corsano, Gianluca Santoro, Maria Clara Cavallini, Adriano Schimmenti

**Affiliations:** ^1^Department of Humanities, Social Sciences and Cultural Industries, University of Parma, Parma, Italy; ^2^Faculty of Human and Social Sciences, UKE – Kore University of Enna, Enna, Italy; ^3^Department of Psychology, Catholic University of Milan, Milan, Italy

**Keywords:** problematic gaming, maladaptive personality traits, alexithymia, psychopathology, cluster analysis

## Abstract

**Background:**

A need exists to increase our understanding of the association between maladaptive personality traits, psychopathological symptoms, game preference, and different types of video game use. In the present study, we used a person-centered approach to identify different subtypes of video game players and we explored how they differ in personality profiles, clinical symptoms, and video game usage.

**Methods:**

We assessed problematic gaming via the nine-item Internet Gaming Disorder Scale and self-reported screen time playing video games in a sample of 366 adolescents and young adult gamers. Participants also completed measures on maladaptive personality domains (Personality Inventory for DSM-5 Brief Form), alexithymia (Toronto Alexithymia Scale—20 items), and psychopathological symptoms (DSM-5 Self-Rated Level 1 Cross-Cutting Symptom Measure) and reported which genre of video games they preferred.

**Results:**

Using a person-centered, cluster-analytic approach, we identified four clusters of video game players (Occasional, Passionate, Preoccupied, and Disordered) presenting peculiar combinations of problematic gaming scores and time spent online playing video games. Non-problematic gamers (Occasional and Passionate) represented the majority of the sample (62.3% of the participants). Highly involved gamers who exhibited excessive screen time playing video games (Disordered gamers) presented the highest level of maladaptive personality traits and psychopathological symptoms, and were characterized by the greatest use of Multiplayer Online Battle Arena (MOBA) games.

**Conclusion:**

These results have clinical implications on suggesting the importance to determining whether or not problematic gaming activities reflect a dysfunctional emotion-focused coping strategy to avoid inner unpleasant emotional or a more generally compromised emotional and social functioning.

## Introduction

In the last two decades, an extensive amount of research has been carried out on Internet addiction disorder (IAD), a presumptive new clinical condition that has proved to be an umbrella term including different Internet-related psychopathologies (Schimmenti et al., 2014a, b; Musetti et al., 2016, 2017; Starcevic and Billieux, 2017). Among the latter, Internet gaming disorder (IGD) was included in Section 3 (“Emerging Measures and Models”) of the fifth edition of Diagnostic and Statistical Manual of Mental Disorders (American Psychiatric Association [APA], 2013), and more recently, WHO experts recommended including Gaming disorder (GD) in the ICD-11, in the section of disorders due to addictive behaviors (World Health Organization, 2018). Actually, problematic gaming is an issue that deserves the utmost attention given the increasing number of video gamers (2.5 billions, according to Newzoo Games, 2016).

However, there is still a lack of consensus on criteria, definition, and conceptualization of problematic gaming patterns as a specific psychiatric disorder (Aarseth et al., 2017; Schimmenti and Starcevic, 2019). In fact, the video game use has been associated with both adjustment (e.g., De Freitas and Griffiths, 2007; Achtman et al., 2008; Hussain and Griffiths, 2008; Zhong, 2011; Giner-Bartolomé et al., 2015) and maladjustment (e.g., Smyth, 2007; Gentile, 2009; Hussain and Griffiths, 2009), so conceptualizing problematic gaming in terms of an addiction to video games might be an oversimplification—or another umbrella term—for a set of heterogeneous activities with different functions and causes (Billieux et al., 2015). As well as for the different uses of the Internet, distinguishing among different uses and, more importantly, different motives and functions of video game uses in an individual’s life is useful (Lee et al., 2017). It would be misleading to consider problematic online behaviors, like video gaming, isolated from the psychosocial context in which these behaviors are situated (Musetti and Corsano, 2018) and from the psychosocial needs that the players try to satisfy (Kardefelt-Winther, 2014a) by taking advantage of the opportunities that the game offers, for example, in terms of satisfying identity needs (Mancini and Sibilla, 2017; Sibilla and Mancini, 2018; Mancini et al., 2019).

According to Kardefelt-Winther (2014a, b) compensatory approach, problematic online gaming could be intended as a dysfunctional coping strategy to satisfy psychological needs and to compensate for psychosocial problems, including exposure to difficult life experiences (Schimmenti and Caretti, 2010) and a perceived discrepancy between actual and ideal self (e.g., Klimmt et al., 2009; Mancini et al., 2019). According to this compensatory approach, an individual could use video games for entertainment or escapism purposes, depending on the degree of life problems experienced. Therefore, gamers with high psychological maladjustment, in comparison to gamers with low psychological maladjustment, should be more likely to play video games to thwart negative feelings, thereby starting a vicious circle and ending up with a higher degree of problematic outcomes. In fact, literature shows that problematic video gaming is associated with a wide range of psychological factors.

### Personality Traits and Problematic Video Gaming

Personality traits are specific patterns of behavior, emotion, and thought that are relatively stable over time and situations. Some personality traits have been positively associated with problematic video gaming: neuroticism (Cao and Su, 2007; Mehroof and Griffiths, 2010; Collins et al., 2012; Dalbudak et al., 2013; Yan et al., 2014; Billieux et al., 2015; Lehenbauer-Baum et al., 2015; Müller et al., 2015; Braun et al., 2016), aggressiveness (Kim et al., 2008; Collins et al., 2012; Braun et al., 2016; impulsivity (Billieux et al., 2015; Norbury and Husain, 2015; Starcevic and Aboujaoude, 2017), psychoticism (Cao and Su, 2007; Dalbudak et al., 2013; Laier et al., 2018), and sensation seeking (Mehroof and Griffiths, 2010; Lorains et al., 2011; Jiménez-Murcia et al., 2014; Hodgins and Holub, 2015; Mestre-Bach et al., 2016). Other personality traits have been negatively associated with problematic video gaming: extraversion (Landers and Lounsbury, 2006; Müller et al., 2015; Öztürk et al., 2015), conscientiousness, and openness (Wang et al., 2015). Generally, research suggests that maladaptive personality traits could be vulnerability factors for developing problematic video gaming (Gervasi et al., 2017). More specifically, researchers who have differentiated between different types of players have found that regular gamers, in contrast to problematic gamers, showed low maladaptive personality traits (Braun et al., 2016).

### Alexithymia and Problematic Video Gaming

Difficulty with emotion regulation could be a general risk factor for developing problematic video game use (Billieux et al., 2011; Gaetan et al., 2016; Estévez et al., 2017; Yen et al., 2018; Blasi et al., 2019). Alexithymia is one of the most studied psychological constructs connected to affect dysregulation. It refers to difficulty identifying and describing feelings and is marked by a concrete, externally oriented, cognitive style (Taylor and Bagby, 2013). The association between alexithymia and addictive behaviors has been extensively investigated (Stasiewicz et al., 2012; Bonnaire et al., 2017). However, the literature on the association between alexithymic characteristics and problematic video game use is still scarce (Baysan-Arslan et al., 2016; Zastrow, 2017). In a recent study, Bonnaire and Baptista (2019) found that being alexithymic almost doubled the risk of being a problematic gamer. Similarly, Maganuco et al. (2019) found that increased difficulty identifying and describing feelings was predictive of an excessive Internet use among video game players. From a psychodynamic perspective it can be stated that, for some individuals who spend time playing video games, problematic gaming could be a coping strategy (albeit dysfunctional) to manage temporary or chronic emotional distress (Seay and Kraut, 2007; Schimmenti and Caretti, 2010; Schimmenti et al., 2012; Blasi et al., 2019; Mancini et al., 2019).

### Psychopathological Symptoms and Problematic Video Gaming

In regard to related psychopathological symptoms, problematic gaming has been associated with a wide number of clinical conditions (Müller et al., 2015) such as depression, ADHD, anxiety, and social phobia (Cole and Hooley, 2013; Hyun et al., 2015; Laconi et al., 2017; Wang et al., 2018), mood and anxiety symptoms (Gentile et al., 2011; Lemola et al., 2011; Mentzoni et al., 2011; Wei et al., 2012; Brunborg et al., 2014; Van Rooij et al., 2014; Lobel et al., 2017), somatic symptoms (Biolcati, 2010), dissociation (Hussain and Griffiths, 2009; Guglielmucci et al., 2019), and suicidal ideation (Rehbein et al., 2010). However, the direction of these associations are not yet clear (Dong et al., 2011).

As recommended in recent literature on problematic gaming and behavioral addictions more generally, it is important not to pathologize common behaviors (Kardefelt-Winther et al., 2017; Tunney and James, 2017; Starcevic et al., 2018). As proof of this, a study of Konkolÿ Thege et al. (2015) showed that several activities often considered behavioral addictions, including problematic video gaming, are frequently context-dependent and transient for most individuals. Therefore, more studies are needed to assess the association between psychopathological symptoms and specific types of problematic and non-problematic gamers (Billieux et al., 2015).

### Video Game Types and Problematic Video Gaming

Different types of video games have different addictive potential (King et al., 2011). Problematic gaming has frequently been associated with specific video game types, namely Multiplayer online role-playing games (MMORPGs) and Multiplayer Online Battle Arena (MOBA). MMORPGs are virtual worlds in which players extensively cooperate to explore the environment, fight enemies and resolve quests. Problematic MMORPGs use has been reported as the most frequent Internet-related problem in some studies (e.g., Thorens et al., 2014), and several studies have also reported that problematic gaming is more frequent in players who use MMORPGs (Ng and Wiemer-Hastings, 2005; Chuang, 2006; Peters and Malesky, 2008; Collins et al., 2012; Eichenbaum et al., 2015a, b; Lemmens et al., 2015).

In MOBA games, two teams composed of different players compete each other. Differently to MMORPGs, MOBA games do not develop in never-ending worlds; yet they provide extensive feedbacks to the players, stimulating competition and social interaction. MOBA games have also been associated with problematic gaming in research (Fuster et al., 2016; Triberti et al., 2018).

The addictive potential of other game genres and typologies is still under debate. While there is some evidence that an excessive use of first-person shooter (FPS) is related to increased clinical symptoms (Na et al., 2017), it remains unclear if other types of video games (e.g., casual games) and single-play video games are associated with psychopathology.

### The Present Study

In this study, we aimed to explore the relationships among maladaptive personality domains, alexithymia, psychopathological symptoms, game preference, and problematic gaming in adolescents and young adults. In fact, individuals within these age groups use video games the most (Griffiths et al., 2004) and more frequently display symptoms of problematic gaming (Gentile, 2009; Kuss and Griffiths, 2012). In line with the literature, we expected positive associations among problematic gaming, time spent online playing video games, psychopathological symptoms, maladaptive personality traits, and alexithymia. However, in the present research we also used a person-centered approach to identify different groups of video game players presenting peculiar combinations of problematic gaming scores and time spent online playing video games.

A person-centered approach treats the person as the unit of analysis, by identifying meaningful subgroups (e.g., clusters) of participants characterized by distinct pattern of relationship on the variables of interest that differentiate them from other subgroups of participants. This allows researchers to relate these clusters to meaningful other variables (e.g., Zuber et al., 2015). According to Bergman and Trost (2006) “a ‘person-oriented’ approach is one in which the focus is to understand development at the individual level by regarding the individual as a functioning whole with processes operating at a system level and its components jointly contributing to what happens in development. By ‘components’, we mean, for example, behaviors, biological factors, perceptions, goals, and values, among other aspects that make up the structure of the individual” (p. 604). In our view, this approach could stimulate the development of a more comprehensive understanding of the association between the behavioral and the psychological components of problematic gaming. Differently from variable-centered approaches which focus on the stability of the variables, person-centered approaches focus on inter-individual differences and similarities among participants, thus providing a complementary perspective on the relationships among variables (Morin and Wang, 2016). By adopting this approach, we aimed to overcome the distinction most classically present in the literature (and frequently based on cut-off scores of measures used for the screening of problematic gaming) between pathological and non-pathological players, and thus to potentially discover different specific subgroups of gamers, whose screen time playing video games and scores on problematic gaming might reflect further differences in personality features, clinical symptoms, and preference for specific video game types.

In line with previous research, we expected to identify a subset of problematic gamers with increased time spent online playing video games and high scores on IGD. Moreover, we expected that the video game players identified as problematic gamers would present higher levels of maladaptive personality traits (Amendola et al., 2019), psychopathological symptoms (Müller et al., 2015), and alexithymia (Bonnaire et al., 2017; Maganuco et al., 2019) than the other players. Finally, we hypothesized that this subgroup of highly involved gamers would use more immersive games such as Multiplayer Online Role-Playing Game (MMORPGs) (Eichenbaum et al., 2015a, b) and Multiplayer Online Battle Arena (MOBA) games (Fuster et al., 2016; Triberti et al., 2018).

## Materials and Methods

### Participants

The study involved 366 Italian adolescents and young adults (311 males, 85%; 55 females, 15%) aged from 15 to 30 years (*M* = 21.64 years, *SD* = 3.77) recruited via an online survey. An advertisement was shared on different video game platforms, such as video game forums, Facebook video game groups and web-pages dedicated to video games. Out of 400 participants who accessed the online survey, 366 (91.5%) accepted to participate and signed the electronic informed consent. All questions in the online survey were mandatory, so there were no missing cases. The socio-demographic characteristics of the sample are described in Table 1.

**TABLE 1:** Socio-demographic characteristics the participants.

		**1**	**2**	**3**	**4**
	***N* = 366**	**Passionate 142**	**Occasional 86**	**Preoccupied 76**	**Disordered 62**
**Gender**					
Males	311(85%)	117(82.4%)	74(86.0%)	68(89.5%)	52(83.9%)
Females	55(15%)	25(17.6%)	12(14.0%)	8(10.5%)	10(16.1%)
**Marital status**					
Not married	319(87.2%)	126(88.7%)	72(83.7%)	65(85.5%)	56(90.3%)
Domestic partner	39(10.7%)	13(9.2%)	11(12.8%)	9(11.8%)	6(9.7%)
Married	7(1.9%)	3(2.1%)	2(2.3%)	2(2.6%)	0(0.0%)
Widow	1(0.3%)	0(0.0%)	1(1.2%)	0(0.0%)	0(0.0%)
**Education**					
Secondary lower education	110(30.1%)	49(34.5%)	21(24.4%)	21(27.6%)	19(30.6%)
Secondary upper education	206(56%)	76(53.5%)	48(55.8%)	48(63.2%)	34(54.8%)
Bachelor’s degree	39(10.7%)	16(11.3%)	11(12.8%)	5(6.6%)	7(11.3%)
Master’s degree	11(3.0%)	1(0.7%)	6(7.0%)	2(2.6%)	2(3.2%)
**Working status**					
Students	194(53.0%)	63(44.4%)	53(61.3%)	43(56.6%)	35(56.5%)
Employees	69(18.9%)	36(25.4%)	14(16.3%)	10(13.2%)	9(14.5%)
Student-workers	46(12.6%)	18(12.7%)	9(10.5%)	15(19.7%)	4(6.5%)
Unemployed	38(10.4%)	19(13.4%)	5(5.8%)	3(3.9%)	11(17.7%)
Freelancers	18(4.9%)	6(4.2%)	4(4.7%)	5(6.6%)	3(4.8%)
Other	1(0.3%)	0(0.0%)	1(1.2%)	0(0.0%)	0(0.0%)

There was no significant association between participants’ gender and age (*t*_(__364__)_ = −1.98, *p* = 0.98) or gender and years of education (*t*_(__364__)_ = −0.09, *p* = 0.77). The mean number of hours spent daily on video games was 3.58 on average (*SD* = 1.79, range 1–10), which is comparable to the findings of other studies (Billieux et al., 2015).

### Procedures

Ethical clearance was obtained from the Internal Review Board for Psychological Research of the UKE–Kore University of Enna. The inclusion criteria were being in the middle or late adolescence or emerging-adulthood life stage (i.e., between 15 and 30 years old), self-identifying as a “gamer”, and reporting no use of psychotropic medications. All of the participants gave their informed consent and completed an anonymous questionnaire containing socio-demographic information (age, gender, years of education, marital status, employment), the number of daily hours they used video games, the type of video games used, and self-reported scales on IGD, maladaptive personality domains, alexithymia, and psychopathological symptoms. Anonymity of the participants was guaranteed (no data on the gamers’ identification were collected, including their Internet Protocol address). Participants did not take any compensation for their involvement in the study. The study was carried out according to the Ethical Code of the Italian Association of Psychology (AIP) and the American Psychological Association (APA).

### Measures

#### Sociodemographics

The questionnaire included questions concerning gender, age, marital status, educational level, and employment to obtain a profile of the respondents’ demographic features.

#### Video Games Genres

Participants were asked to indicate (yes/no) which genre of video games they play among Massively Multiplayer Online Role-Playing Games (MMORPG), Multiplayer Online Battle Arena (MOBA), browser games, first-person shooter (FPS) games, real time strategy (RTS) games, or simulation games.

#### Internet Gaming Disorder

The Italian nine-item Internet Gaming Disorder Scale (IGD-9; Pontes and Griffiths, 2015; Italian version by Monacis et al., 2016) was used to assess the severity of IGD by examining gaming activities occurring over a 12-month period. The scale includes nine items corresponding to the nine core criteria defined by the DSM-5 (American Psychiatric Association [APA], 2013). Subjects were asked to answer on a 5-point Likert-type scale ranging from 1 (never) to 5 (very often), rather than on the original scale comprising dichotomous items (yes/no), to improve the psychometric properties and reliability of the assessment (Comrey, 1988; Haladyna, 1992). An examples of IGD-9 items is “Have you lost interest in previous hobbies and other entertainment activities as a result of your engagement with the games?”. Higher scores indicate higher symptoms of IGD. Cronbach’s alpha of the IGD-9 in this study was.79.

#### Personality Domains

The Italian version of the Personality Inventory for DSM-5 Brief Form (PID-5-BF; Krueger et al., 2012; Italian adaptation by Fossati et al., 2013) was administered to assess participants’ personality features. The PID-5-BF includes 25 items assessing five maladaptive personality domains: negative affect (5 items; e.g., “I fear being alone in life more than anything else”; Cronbach’s α = 0.61); detachment (5 items; e.g., “I often feel like nothing I do really matters”; Cronbach’s α = 0.69); antagonism (5 items; e.g., “It’s no big deal if I hurt other peoples’ feelings”; Cronbach’s α = 0.66); disinhibition (5 items; e.g., “I feel like I act totally on impulse”; Cronbach’s α = 0.56); and psychoticism (5 items; e.g., “I have seen things that weren’t really there”; Cronbach’s α = 0.68). Items are rated on a 4-point Likert scale ranging from 0 (very false or often false) to 3 (very true or often true). The higher the score, the more dysfunctional the individual’s personality is. The Cronbach’s alpha for the PID-5-BF total score in this study was.81.

#### Alexithymia

The Italian Toronto Alexithymia Scale (TAS-20; Bagby et al., 1994; Italian version by Bressi et al., 1996) was administered to assess alexithymia. The TAS-20 includes 20 items rated on a 5-point Likert-type scale, and responses range from strongly disagree (1) to strongly agree (5). Total scores range from 20 to 100, with higher scores indicating higher levels of alexithymia. The TAS-20 has a three-factor structure (Haviland, 1996): difficulty identifying feelings (DIF; 7 items; e.g., “I am often confused about what emotion I am feeling”; Cronbach’s α = 0.81); difficulty describing feelings (DDF; 5 items; e.g., “It is difficult for me to find the right words for my feelings”; Cronbach’s α = 0.77); and externally oriented thinking (EOT; 8 items; e.g., “I prefer talking to people about their daily activities rather than their feelings” Cronbach’s α = 0.53), which was also confirmed in adolescent populations (Säkkinen et al., 2007; Parker et al., 2010). In this study, the Cronbach’s alpha of the entire TAS-20 scale was.79.

#### Clinical Symptoms

The DSM-5 Self-Rated Level 1 Cross-Cutting Symptom Measure (American Psychiatric Association [APA], 2013) was used to assess psychopathological symptoms. This measure includes 23 items rated on a 4-point Likert-type scale ranging from 0 (very false or often false) to 3 (very true or often true). Each item investigates how often an individual has suffered from specific symptoms in the last 2 weeks. The following are the 13 psychopathological domains: depression (2 items; e.g., “Feeling down, depressed, or hopeless”; Cronbach’s α = 0.70); anger (1 item: “Feeling more irritated, grouchy, or angry than usual?”); mania (2 items; e.g., “Sleeping less than usual, but still have a lot of energy?”; Cronbach’s α = 0.39); anxiety (3 items; e.g., “Feeling panic or being frightened?” Cronbach’s α = 0.61); somatic symptoms (2 items; e.g., “Feeling that your illnesses are not being taken seriously enough?”; Cronbach’s α = 0.67); suicidal ideation (1 item: “Thoughts of actually hurting yourself?”); psychosis (2 items; e.g., “Hearing things other people couldn’t hear, such as voices even when no one was around?”; Cronbach’s α = 0.36); sleep problems (1 item: “Problems with sleep that affected your sleep quality over all?); memory (1 item: “Problems with memory [e.g., learning new information] or with location [e.g., finding your way home]?”); repetitive thoughts and behaviors (2 items; e.g., “Unpleasant thoughts, urges, or images that repeatedly enter your mind?”; Cronbach’s α = 0.64); dissociation (1 item: “Feeling detached or distant from yourself, your body, your physical surroundings, or your memories?”); personality functioning (2 items; e.g., “Not feeling close to other people or enjoying your relationships with them?”; Cronbach’s α = 0.64); substance use (3 items; e.g., “Using any of the following medicines on your own, that is, without a doctor’s prescription, in greater amounts or longer than prescribed [e.g., painkillers (like Vicodin), stimulants (like Ritalin or Adderall), sedatives or tranquilizers (like sleeping pills or Valium), or drugs like marijuana, cocaine or crack, club drugs (like ecstasy), hallucinogens (like LSD), heroin, inhalants or solvents (like glue), or methamphetamine (like speed)]?”; Cronbach’s α = 0.61).

### Data Analyses

Data analyses were aimed to (1) describing video game players’ socio-demographic carachteristics, personality functioning and psychopathological symptoms, (2) identifying clusters of players based on hours spent on video games and IGD-9 scores, and (3) examining how personality functioning and psychopathological symptoms were related to cluster membership.

Descriptive statistics and Pearson’s correlation analysis were performed to explore the associations among the investigated variables. Considering that Pearson’s correlation analysis was conducted with a wide number of tests, the Holm’s method was used to correct for multiple statistical comparisons. Subsequently, in order to identify subgroups of players similar on IGD scores and on time spent to play with video games (i.e., in order to apply a person-centered approach to the collected data collected), we performed a two-step cluster analysis. This is an exploratory procedure that allows researchers to identify data-driven subgroups (clusters) within sufficiently large data set (i.e., N ≥ 250; Norušis, 2010). This analysis follows two steps. First, it identifies groups with a clustering algorithm based on a distance measure between the rough scores of the variables. Second, it automatically selects the optimal number of clusters by applying hierarchical methods. A *p*-value of 0.05 was set as the critical level for statistical significance. As in other studies that used this statistical procedure (e.g., Schimmenti, 2016), a log-likelihood distance measure with Schwarz Bayesian information criterion was used to determine the number of clusters and establish a data-driven classification of video game players. Silhouette measure of cohesion and separation was evaluated as a measure of validity of the within- and between-cluster distances.

Finally, in order to analyze associations between sociodemographic variables, maladaptive personality domains, alexithymia, psychopathological symptoms, and game preferences in the identified clusters, a series of one-way ANOVAs and Chi–square tests were performed using the clusters as factors. The cluster differences were assessed using *post hoc* Bonferroni test (for ANOVAs) or cell residuals (for Chi–square tests).

## Results

### Descriptive Statistics

Descriptive statistics of the sample (*N* = 366) are shown in Table 2. Video game players in this sample mostly preferred FPS, MOBA and MMORPG games. They reported increased scores on clinical symptoms and severe alexithymic traits on average.

**TABLE 2:** Descriptive statistics.

	**Frequencies**	**Percentage**	
**Preferred games**			
MMORPG	197	53.8%	
MOBA	196	53.6%	
Browser games	46	12.6%	
FPS	236	64.5%	
RTS	80	21.9%	
Simulation	166	45.4%	
Spend money for in-game features	190	51.9%	

	**Mean**	**Standard deviation**	**Observed range**

Age	21.64	3.77	15–30
Hours per day spent in videogames	3.58	1.79	1–10
IGD-9	17.40	5.77	9–39
PID-5-BF	24.55	10.08	0–53
Negative affectivity	7.12	3.15	0–14
Detachment	4.75	3.26	0–14
Antagonism	3.67	2.96	0–14
Disinhibition	4.52	2.77	0–13
Psychoticism	4.48	3.00	0–15
TAS-20	70.42	11.66	44–97
Difficulty identifying feelings	26.90	6.03	10–35
Difficulty describing feelings	15.05	4.96	5–25
Externally oriented thinking	28.46	4.86	13–40
Level one	2.69	0.55	0–2.69
Depression	1.47	1.03	0–4
Anger	1.28	1.20	0–4
Mania	1.04	0.93	0–4
Anxiety	1.12	0.92	0–4
Somatic symptoms	0.96	1.08	0–4
Suicidal ideation	0.33	0.84	0–4
Psychosis	0.17	0.45	0–3
Sleep problems	1.02	1.25	0–4
Memory	0.45	0.86	0–4
Repetitive thoughts and behaviors	0.68	0.94	0–4
Dissociation	0.51	0.94	0–4
Personality functioning	1.25	1.10	0–4
Substance use	0.60	0.86	0–4

### Associations Between Variables

A pattern of significant and positive associations emerged among IGD-9 scores, time spent online playing video games (*r* = 0.31, *p* < 0.001), maladaptive personality traits (*r_*PID–*__5__–TOT_* = 0.27, *p* < 0.001), and psychopathological symptoms (*r_*DSM–*__5__–LEVEL__1_* = 0.38, *p* < 0.001). Unexpectedly, a pattern of significant and negative associations was observed in the relationship between alexithymia and IGD-9 scores (*r_*TAS–*__20_* = -0.22, *p* < 0.01), maladaptive personality traits (*r_*PID–*__5__–TOT_* = -0.54, *p* < 0.01), and psychopathological symptoms (*r_*DSM–*__5__–LEVEL__1_* = -0.47, *p* < 0.001). Supplementary Table S1 display the correlations among all the investigated variables.

### Clusters of Video Game Players

The two-step cluster analysis generated four clusters. The average silhouette measure value of cohesion and separation was 0.5, which is considered a fair to good solution for discriminating the groups (Rousseeuw and Kaufman, 1990).

The first cluster was characterized by a relatively high amount of time spent on video games (*M* = 4.01 h per day, *SD* = 1.02) but a non-problematic gaming use score (*M*_*IGD–*__9_ = 13.94, *SD*_*IGD–*__9_ = 2.09), so we used labeled them Passionate gamers. This was the significantly largest group identified by cluster analysis (*N* = 142; 38.8%), χ*2*(3) = 40.34, *p* < 0.01.

The second cluster was labeled Occasional gamers (*N* = 86; 23.5%) because its members used video games for a low amount of time (*M* = 1.62 h per day, *SD* = 0.49) and had non-problematic gaming use (*M*_*IGD–*__9_ = 13.76, *SD*_*IGD–*__9_ = 2.66).

We labeled the members of the third cluster as Preoccupied gamers (*N* = 76; 20.8%), because they played an average number of hours on video games (*M* = 2.92 h per day, *SD* = 0.80) but reported high levels of preoccupations and symptoms concerning their game use (*M*_*IGD–*__9_ = 21.75, *SD*_*IGD–*__9_ = 2.82).

We labeled the members of the fourth cluster as Disordered gamers (*N* = 62; 16.9%) because they were characterized by a very high amount of time on video games (*M* = 6.13 h per day, *SD* = 1.62) and reported severe symptoms of problematic game use (*M*_*IGD–*__9_ = 25.06, *SD*_*IGD–*__9_ = 6.29). The four clusters did not differ with respect to gender, χ*2*(3) = 2.08, *p* = 0.56; age, F(3, 262) = 0.49, *p* = 0.69; and years of education, F(3, 262) = 1.64, *p* = 0.18.

### Differences Between Clusters

Descriptive statistics for each cluster and the ANOVA results (including results of Bonferroni’s *post hoc* analyses) are reported in Table 3.

**TABLE 3:** Differences between clusters of videogame players on IGD-9 scores, time spent playing videogames, age, years of education, maladaptive personality traits, alexithymia, and clinical symptoms.

		**Clusters**				**ANOVA**		
		**1**	**2**	**3**	**4**			
		**Passionate**	**Occasional**	**Preoccupied**	**Disordered**	***F(3, 362)***	***p***	**Bonferroni tests**
*N*		142	86	76	62			
IGD-9	*M*	13.94	13.76	21.75	25.06	226.07	0.000	4 > 1, 2, 3; 3 > 1, 2
	*(SD)*	(2.09)	(2.66)	(2.82)	(6.29)			
	*Observed range*	9-19	9-18	18-29	13-39			
Hours per day on videogames	*M*	4.01	1.62	2.92	6.13	254.73	0.000	4 > 1 > 3 > 2
	*(SD)*	(1.02)	(0.49)	(0.80)	(1.62)			
	*Observed range*	3-7	1-2	1-4	3-10			
Age	*M*	21.48	21.80	21.41	22.06	0.49	0.69	-
	*(SD)*	(3.83)	(4.18)	(3.61)	(3.25)			
Years of education	*M*	11.65	12.51	11.95	11.97	1.64	0.18	-
	*(SD)*	(2.84)	(2.98)	(2.67)	(2.92)			
PID-total score	*M*	0.87	0.89	1.11	1.20	15.66	0.000	4 > 2, 1; 3 > 2, 1
	*(SD)*	(0.37)	(0.40)	(0.37)	(0.40)			
PID-negative affect	*M*	1.29	1.30	1.63	1.64	8.76	0.000	4 > 2, 1; 3 > 2, 1
	*(SD)*	(0.58)	(0.63)	(0.59)	(0.66)			
PID-Detachment	*M*	0.82	0.85	1.05	1.26	8.37	0.000	4 > 1, 2
	*(SD)*	(0.64)	(0.62)	(0.64)	(0.63)			
PID-Antagonism	*M*	0.60	0.72	0.83	0.94	5.86	0.001	4 > 1; 3 > 1
	*(SD)*	(0.59)	(0.53)	(0.56)	(0.65)			
PID-Disinhibition	*M*	0.85	0.79	1.04	1.01	3.96	0.008	3 > 2
	*(SD)*	(0.56)	(0.51)	(0.54)	(0.58)			
PID-Psychoticism	*M*	0.78	0.79	1.01	1.17	8.05	0.000	4 > 1, 2; 3 > 1
	*(SD)*	(0.54)	(0.60)	(0.55)	(0.69)			
TAS-20 total score	*M*	72.92	72.24	65.82	67.79	8.35	0.000	1 > 4, 3; 2 > 3
	*(SD)*	(11.38)	(10.77)	(10.78)	(12.52)			
TAS-20 DIF	*M*	28.60	27.49	24.41	25.23	10.74	0.000	1 > 4, 3; 2 > 3
	*(SD)*	(5.39)	(5.90)	(5.60)	(6.75)			
TAS-20 DDF	*M*	15.82	15.50	13.80	14.21	3.66	0.01	1 > 3
	*(SD)*	(5.36)	(4.75)	(4.24)	(4.78)			
TAS-20 EOT	*M*	28.49	29.26	27.61	28.35	1.57	0.20	-
	*(SD)*	(4.79)	(4.91)	(4.81)	(4.95)			
Depression	*M*	1.19	1.27	1.83	1.98	14.12	0.000	4 > 2, 1; 3 > 2, 1
	*(SD)*	(0.90)	(0.95)	(1.02)	(1.16)			
Anger	*M*	1.07	1.03	1.66	1.66	7.59	0.000	4 > 2, 1; 3 > 2, 1
	*(SD)*	(1.08)	(1.12)	(1.25)	(1.34)			
Mania	*M*	1.03	1.02	0.97	1.21	0.86	0.46	-
	*(SD)*	(0.96)	(0.96)	(0.72)	(1.04)			
Anxiety	*M*	0.92	1.00	1.46	1.31	7.45	0.000	3 > 2, 1; 4 > 1
	*(SD)*	(0.87)	(0.94)	(0.91)	(0.86)			
Somatic Symptoms	*M*	0.85	0.80	1.11	1.21	2.82	0.04	−
	*(SD)*	(1.03)	(1.04)	(1.18)	(1.08)			
Suicidal Ideation	*M*	0.22	0.29	0.33	0.61	3.29	0.02	4 > 1
	*(SD)*	(0.74)	(0.75)	(0.79)	(1.15)			
Psychosis	*M*	0.11	0.13	0.19	0.36	5.15	0.00	4 > 2, 1
	*(SD)*	(0.31)	(0.33)	(0.47)	(0.73)			
Sleep Problems	*M*	0.90	0.86	1.16	1.31	2.30	0.08	−
	*(SD)*	(1.19)	(1.08)	(1.38)	(1.41)			
Memory	*M*	0.37	0.36	0.49	0.68	2.21	0.09	−
	*(SD)*	(0.78)	(0.80)	(0.79)	(1.13)			
Repetitive Thoughts and Behaviors	*M*	0.56	0.60	0.78	0.93	2.82	0.04	−
	*(SD)*	(0.89)	(0.80)	(1.07)	(1.02)			
Dissociation	*M*	0.42	0.35	0.66	0.74	3.27	0.02	−
	*(SD)*	(0.93)	(0.78)	(0.93)	(1.10)			
Personality Functioning	*M*	0.93	1.06	1.43	2.01	17.30	0.000	4 > 3, 2, 1; 3 > 2, 1
	*(SD)*	(0.98)	(0.94)	(1.03)	(1.25)			
Substance Use	*M*	0.61	0.49	0.78	0.51	1.75	0.16	−
	*(SD)*	(0.90)	(0.68)	(0.97)	(0.87)			

The ANOVA results showed statistically significant differences between the clusters for the following variables: PID-total score, *p* < 0.01; PID-negative affect, *p* < 0.01; PID-detachment, *p* < 0.01; PID-antagonism, *p* < 0.01; PID-disinhibition, *p* < 0.01; PID-psychoticism, *p* < 0.01; TAS-20 total score, *p* < 0.01; TAS-20 DIF, *p* < 0.01; TAS-20 DDF, *p* < 0.05; depression, *p* < 0.01; anger, *p* < 0.01; anxiety, *p* < 0.01; suicidal ideation, *p* < 0.05; psychosis, *p* < 0.01; personality functioning, *p* < 0.01. Bonferroni’s *post hoc* tests were performed to assess the differences between the groups. In regard to maladaptive personality traits, Disordered gamers and Preoccupied gamers showed significantly higher PID-5 total scores and scores on negative affect than Occasional gamers and Passionate gamers, and significantly higher psychoticism and antagonism than Passionate gamers. Disordered gamers showed significantly higher detachment than Occasional gamers and Passionate gamers, and significantly higher psychoticism than Occasional gamers. Preoccupied gamers showed significantly higher disinhibition than Occasional gamers.

Concerning alexithymia, surprisingly, Passionate gamers showed significantly higher TAS-20 total score and significantly higher difficulty identifying feelings scores than Disordered gamers and Preoccupied gamers, and significantly higher difficulty describing feelings than Preoccupied gamers. Occasional gamers showed significantly higher TAS-20 total scores and significantly higher difficulty identifying feelings than Preoccupied gamers. However, it should be noted that all the four clusters showed mean scores above the TAS-20 cutoff of 61 points, revealing high levels of alexithymia (Bagby et al., 1994) in our total sample. Therefore, alexithymia can be considered a common characteristic shared by the group of participants of the present research.

With regard to clinical symptoms, Disordered gamers and Preoccupied gamers showed significantly higher depression and anger than Occasional gamers and Passionate gamers and significantly higher anxiety than Passionate gamers. Preoccupied gamers also showed significantly higher anxiety than Occasional gamers. Disordered gamers showed significantly higher suicidal ideation than Passionate gamers and significantly higher psychosis than Passionate gamers and Occasional gamers. Disordered gamers showed significantly higher disordered personality functioning than the other clusters, and Preoccupied gamers showed a significantly higher disordered personality functioning than Occasional gamers and Passionate gamers. Disordered gamers showed the highest scores for dissociation, but *post hoc* analyses did not reveal specific differences between groups for this variable.

Subsequently, we explored the video games use (preferred genres of games) by the gamers. Frequencies of variables of interest are shown in Table 4. Disordered gamers showed the highest use of MOBA games, χ*2*(3) = 14.44, *p* < 0.01, and a moderate, at-the-limit-of-significance use of MMORPG games, χ*2*(3) = 7.61, *p* = 0.05, with respect to other players.

**TABLE 4:** Genre of the video games used by the four cluster players.

		**Clusters**				**Chi-square**	
		**1**	**2**	**3**	**4**		
		**Passionate**	**Occasional**	**Preoccupied**	**Disordered**	**χ*^2^(3)***	***p***
*N*		142	86	76	62		
MMORPG	*N*	83	36	40	38	7.61	0.05
	*(%)*	(58.5%)	(41.9%)	(52.6%)	(61.3%)		
MOBA	*N*	76	34	42	44	14.74	0.00
	*(%)*	(53.5)	(39.5)	(55.3%)	(71%)		
Browser games	*N*	16	11	9	10	9.74	0.81
	*(%)*	(11.3%)	(12.8%)	(11.8%)	(16.1%)		
FPS	*N*	94	52	47	43	1.66	0.64
	*(%)*	(66.2%)	(60.5%)	(61.8%)	(69.4%)		
RTS	*N*	25	22	17	16	2.78	0.43
	*(%)*	(17.6%)	(25.6%)	(22.4%)	(25.8%)		
Simulation	*N*	67	37	38	24	2.15	0.54
	*(SD)*	(47.2%)	(43%)	(50%)	(38.7%)		

## Discussion

The first aim of the current study was to explore the associations among problematic gaming, time spent online playing video games, psychopathological symptoms, maladaptive personality traits, and alexithymia in a group of adolescent and young adult video game players. In line with the literature, a pattern of positive associations was found among problematic gaming, time spent online playing video games, psychopathological symptoms, and maladaptive personality traits. Unexpectedly, alexithymia scores were negatively associated with IGD-9 scores, psychopathological symptoms, and maladaptive personality traits.

Subsequently, we aimed to identify different subtypes of video game players presenting peculiar combinations of IGD-9 scores and times of video game use, and to explore the link between different profiles of video gamers and psychological maladjustment.

Two clusters (Occasional gamers and Passionate gamers) likely included non-problematic gamers and represented the majority of the sample (62.3% of the participants). These gamers showed IGD-9 scores below the Italian cutoff point of 21 used to determine the presence of problematic gaming use (Monacis et al., 2016), but only Passionate gamers spent a large amount of time on video games, e.g., according to Kim et al. (2016) more than 4 h per day.

In line with the literature, both kinds of non-problematic players presented low levels of psychological maladjustment (maladaptive personality traits and psychopathological symptoms) regardless of the number of hours spent daily on video games, with respect to the other identified clusters. This result is in line with literature stating that high involvement in gaming is not problematic *per se* (Charlton and Danforth, 2007), but it can represent a passionate use (Billieux et al., 2013; Burnay et al., 2015; Deleuze et al., 2018; Sibilla, 2019). The first cluster included the largest subtype of gamers in our study, i.e., the Passionate gamers. As shown by previous studies (Sibilla and Mancini, 2018), it is likely that passionate gamers do not play to satisfy specific psychological needs, for example those related to identity needs (Mancini and Sibilla, 2017; Sibilla and Mancini, 2018; Mancini et al., 2019). For them, playing could simply represent a playful activity even if it is demanding in terms of time, but without negative implications on their psychological processes. However, contrary to what was expected, members of this cluster showed the highest level of alexithymia. It is thus possible that these gamers, who spent many hours of their free time using video games, are oriented toward actions and the mission of the game, i.e., in achievement motives (Yee, 2006) rather than on gaming motives associated with self-reflection and introspection (Maganuco et al., 2019). On the other hand, having a poor capacity to identify and describe emotions is a characteristic of all the identified clusters. This result is in line with research by Gaetan et al. (2016), who found that regular gamers have more difficulty identifying and expressing emotions and suggested that video game environments may function as a tool to “curb alexithymic dynamic” (p. 347), transforming chaotic emotions into psychologically meaningful events.

The second subtype of non-problematic gamers is composed of Occasional video game players, who present even less psychological maladjustment, in comparison to Passionate gamers. This subgroup of gamers probably includes those individuals who consider video games as a hobby among others. However, it should be noted that even in this case, high levels of alexithymia were reported. This result must be taken with caution because in the present research we have not taken into consideration non-players of the same cohort. Therefore, other studies are needed to investigate alexithymia in low- and high-engaged gamers.

The other two groups identified by cluster analysis appear as more problematic. Both clusters of gamers showed IGD-9 scores over the Italian cutoff point of 21 used to determine the presence of problematic gaming (Monacis et al., 2016), but only Disordered gamers spent a very high amount of screen time playing video games, by using the cut-off value proposed by Kim et al. (2016) of more than 6 h per day to identify problematic gamers. The third cluster is that of Preoccupied gamers. The members of this group showed high levels of maladjustment although they did not spend a lot of time on video games. Like all the other players in our sample, they were alexithymic on average; moreover, they also presented the highest levels of anxiety, and higher anger and depression than Occasional and Passionate gamers. It is possible that this combination of symptoms describes their difficulty facing a great deal of negative affects without having sufficient capacity for emotional regulation. So, they could represent a group of individuals who display clinical symptoms that are independent of video game use and who play to cope with emotional distress, rather than for an intrinsic motivation to succeed or for leisure (Billieux et al., 2013).

Finally, the last group was composed of Disordered gamers. As expected, these video game players presented the highest level of psychological maladjustment. In particular, they showed the most compromised personality functioning characterized by high levels of psychoticism, psychotic symptoms and suicidal ideation: an overall severe clinical picture that seems to suggest that the virtual world of video games for some of these players could be taken as an alternative to a real social world, as a sort of a “psychic pit” paradoxically protecting these individuals from distressing and perhaps disordered mental states (Schimmenti and Caretti, 2010; Schimmenti et al., 2012). In other words, Disordered gamers could play more to escape from an unbearable real life than to succeed in the game (Deleuze et al., 2019). In addition, the relationship among escapism, psychopathological symptoms and gaming is well known in the literature (Király et al., 2015). This cluster is similar to a cluster of video game players previously described as the “unregulated escapers” by Billieux et al. (2015), as well as with the cluster labeled Escapist that was found by Schuurman et al. (2008) in their study on video game players, which included individuals for whom escapism was the main motive for playing.

Regarding to the genre of video game used, Disordered gamers are characterized by the highest use of MOBAs and by a moderate, at-the-limit-of-significance use of MMORPGs with respect to other players. This may be explained with the fact that by providing very frequent feedback and updates on international rankings and statistics, MOBA games currently involves much more players than MMORPGs (Bonnaire and Baptista, 2019). On the other hand, MOBAs and MMORPGs, being massive and multiplayer games, have generally more immersive features than other video games. Thus, there could be a relationship between the specific characteristics of the digital environment and the problematic use of video games. Other studies are needed to better explore this relationship, for example, studies that account for the motives for playing specific video games, or studies that explore the role that types of games (multiplayers or single player) or types of avatars (e.g., humanoid or non-humanoid avatars) can play for the disordered gamers. In the light of the current literature and of the results of the present study, it can be hypothesized is that disordered gamers somewhat replace a part of their offline social reality, which is likely full of psychological problems and which is perhaps not responsive to important psychosocial needs (Deleuze et al., 2019), with a specific digital reality that provides a high rate of recognition of their activity and their skill as a gamer.

As with every research, the present study comes with a number of limitations. First, the high percentage of male participants and the limited sample size must be highlighted to avoid generalizations. Furthermore, we used a convenient sample, by recruiting video game players in gaming web-pages and forums so, there is a need to replicate these findings in larger groups of gamers. Second, it is acknowledged that collecting clinical information by means of self-report measures can present relevant bias problems (Podsakoff et al., 2003), although the tools used in this study have displayed good psychometric properties in worldwide research. Probably, a multimethod assessment of personality domains, psychopathological symptoms and gaming use would have led to more valid and reliable findings. Third, Internet Gaming Disorder (IGD) is still a clinical condition in need of further study (American Psychiatric Association [APA], 2013) and its conceptualization and measurement should be further examined and broaden (Van Rooij et al., 2017; Schimmenti and Starcevic, 2019). Qualitative studies (i.e., based on clinical interviews) are needed to gain a better understanding of the nature of problematic gaming. Moreover, specific assessment tools should be built in order not to confuse problematic gaming with high immersion and involvement in the play. Fourth, the cross-sectional design of the study made it impossible to definitively determine the direction of the associations between data and cannot allow us to exclude the possibility that our results were affected by third variables not included in this study (e.g., traumatic experiences, insecure attachment, identity needs). Therefore, longitudinal studies are greatly needed to advance this line of work.

However, despite these limitations, our cross-sectional findings support the hypothesis that a multidimensional perspective on different type of gaming behaviors may be particularly informative for clinicians dealing with individuals who display problematic video game use. Our findings showed two subgroups of video game players who showed problematic gaming. Highly involved gamers who exhibited excessive screen time playing video games presented the highest level of maladaptive personality traits and psychopathological symptoms. Highly involved gamers who exhibited an average screen time playing video games presented a high level of negative affectivity (anxiety, anger, and depression). Therefore, it appears to be important to determine whether or not problematic gaming activities reflect a dysfunctional emotion-focused coping strategy to avoid inner unpleasant emotional states (e.g., shelter the self from emotional “high voltage”) or a more generally compromised emotional and social functioning (e.g., absorption in the alternative reality of video games to escape from psychosocial stressors). This distinction could help clinicians understand the factors underlying problematic gaming patterns, and this may foster tailored psychological interventions for people with problematic gaming. In fact, our findings suggest that the assessment of the psychological and psychopathological factors underlying problematic gaming could be more informative than an assessment procedure that solely focuses on the likelihood of a diagnosis (e.g., by the use of cut-off scores of measures for the screening of problematic gaming). For example, a preoccupied video game player could benefit from interventions designed to improve emotion regulation and the capacity to reflect on his or her own mental states (e.g., Mentalization-Based Treatment). In contrast, a disordered gamer could benefit from interventions that focus on disordered personality functioning (e.g., Transference-Focused Psychotherapy in the psychodynamic tradition, or Dialectic Behavior Therapy in the cognitive tradition) on one side, and on problematic gaming symptoms on the other side (e.g., behavioral modification techniques, pharmacological treatment) on the other side. Thus, the investigation of the psychological problems and needs of individuals who display excessive game use via a person-centered approach represents a critical opportunity for increasing the scientific understanding of problematic gaming behaviors and, ultimately, for developing tailored and effective treatment.

## Data Availability Statement

The datasets generated for this study are available on request to the corresponding author.

## Ethics Statement

The studies involving human participants were reviewed and approved by the Internal Review Board for Psychological Research of the UKE–Kore University of Enna. Written informed consent to participate in this study was provided by participant or their parent or guardian.

## Author Contributions

AM provided substantial contributions to the conception of the work, deep analysis of the literature, study design, development, and final approval of the manuscript. TM and PC contributed to the development and revision of the work with deep literature analysis and agreement for final approval of the manuscript. GS contributed to the development of the work, with literature review, data acquisition, and agreement for final approval of the manuscript. MC contributed to the revision of the work and agreement for final approval of the manuscript. AS contributed to the conception and deep revision of the work, with literature analysis, contribution to data analysis, and agreement for final approval of the manuscript.

## Conflict of Interest

The authors declare that the research was conducted in the absence of any commercial or financial relationships that could be construed as a potential conflict of interest.
