# Supplementary material for: Maladaptive Personality Functioning and Psychopathological Symptoms in Problematic Video Game Players: A Person-Centered Approach
Source: Front Psychol. 2019 Nov 19;10:2559. doi: 10.3389/fpsyg.2019.02559 (PMC6877750; doi:10.3389/fpsyg.2019.02559)
Supplement: Supplementary file 1 [file Table_1.docx]

| **Table S1.** *Pearson’s r correlations among the investigated variables* | | | | | | | | | | | | | | | | | | | | | | | | | | | |
| --- | --- | --- | --- | --- | --- | --- | --- | --- | --- | --- | --- | --- | --- | --- | --- | --- | --- | --- | --- | --- | --- | --- | --- | --- | --- | --- | --- |
|  | 2 | 3 | 4 | 5 | 6 | 7 | 8 | 9 | 10 | 11 |  | 12 | 13 | 14 | 15 | 16 | 17 | 18 | 19 | 20 | 21 | 22 | 23 | 24 | 25 | 26 | 27 |
| 1. Age | .00 | .08 | -.12 | -.04 | -.09 | -.10 | -.13 | -.14 | .17 | .14 |  | .13 | .20 | .00 | .00 | -.01 | .01 | .01 | 0 | -.01 | -.07 | .10 | .01 | -.09 | -.11 | -.05 | .16 |
| 2. Time spent online playing videogames | - | .31*** | .14 | .12 | .13 | .03 | .09 | .10 | .01 | -.01 |  | .01 | .01 | -.10 | .10 | .08 | .08 | .02 | .03 | .05 | .11 | .03 | .05 | .00 | -.08 | .11* | -.01 |
| 3. IGD-9 |  | - | -.39*** | .27*** | .29*** | .28*** | .16 | .29*** | -.22** | -.27*** |  | -.17 | -.05 | .38*** | .37*** | .28*** | .06 | .24** | .21** | .22** | .19* | .13 | .16 | .22** | .20* | .42*** | -.01 |
| 4. PID-5 (total score) |  |  | - | .64** | .67** | .63** | .60** | .77** | -.54** | -.53** |  | -.43** | -.21** | .58*** | .50*** | .37*** | .21** | .45*** | .31*** | .28*** | .28*** | .16 | .39*** | .43*** | .30*** | .54** | .08 |
| 5. Negative Affect |  |  |  | - | .32*** | .19** | .23** | .36*** | -.42*** | -.31*** |  | -.42*** | -.20 | .44** | .41** | .39** | .11 | .48** | .23** | .20* | .08 | .19* | .26** | .29** | .19* | .32** | -.04 |
| 6. Detachment  7. Antagonism  8. Disinhibition |  |  |  |  | - | .27***  - | .19*  .26***  - | .39***  .41***  .40  *** | -.42***  -.23***  -.33*** | -.31***  -.23***  -.30*** |  | -.42***  -.16  -.21** | -.20*  -.10  -.20* | .40***  .30***  .28*** | .42***  .17  .20* | .22**  .16  .21* | .02  .19*  .19* | .29**  .19*  .17 | .17  .18  .16 | .25***  .11  .13 | .15  .20*  .13 | .10  .05  .03 | .37***  .19*  .12 | .24***  .28***  .24*** | .24**  .10  .08 | .49***  .31***  .22* | -.06  .13  .19* |
| 9. Psychoticism |  |  |  |  |  |  |  | - | -.43*** | -.45*** |  | -.31*** | -.16 | .49*** | .27*** | .26*** | .20* | .35*** | .28*** | .23** | .38*** | .15 | .33*** | .39*** | .37*** | .45*** | .07 |
| 10. TAS-20 (total score)  11. Difficulty Identifying Feelings |  |  |  |  |  |  |  |  | - | .77**  - |  | .81**  .47** | .62**  .12 | -.47***  -.62*** | -.37***  -48*** | -.36***  -.43*** | -.05  -.14 | -.36***  -.46*** | -.28**  -.34*** | -.26**  -.38*** | -.22*  -.28*** | -.16  -.24*** | -.27***  -.31*** | -.30***  -.44*** | -.26***  -.37*** | -.47***  -.51*** | -.09  -.16 |
| 12. Difficulty Describing Feelings |  |  |  |  |  |  |  |  |  |  |  | - | .33*** | -.30*** | -.26** | -.23** | -.04 | -.24*** | -.12 | -.13 | -.07 | -.11 | -.18 | -.17 | -.19* | -.37*** | .01 |
| 13. Externally Oriented Thinking  14. Level-one (total score)  15. Depression |  |  |  |  |  |  |  |  |  |  |  |  | - | -.06  - | -.03  .  68**  - | -.10  .65**  .57** | .10  .  33**  .02 | -.04  .  69**  .47** | -.13  .61**  .33*** | -.02  .  61**  .40*** | -.11  .43**  .13 | .04  .56**  .32** | -.08  .54**  .35*** | .00  .66**  .35*** | .04  .55**  .28*** | -.10  .69**  .53*** | -.03  .28**  .06 |
| 16. Anger |  |  |  |  |  |  |  |  |  |  |  |  |  |  |  | - | .10 | .53*** | .36*** | .31*** | .16 | .26*** | .28*** | .29*** | .22** | .40*** | .05 |
| 17. Mania |  |  |  |  |  |  |  |  |  |  |  |  |  |  |  |  | - | .25** | .14 | .12 | .07 | .17 | .05 | .19** | .15* | .07 | .11 |
| 18. Anxiety |  |  |  |  |  |  |  |  |  |  |  |  |  |  |  |  |  | - | .34** | .42** | .14 | .35*** | .31*** | .43*** | .34*** | .40*** | .05 |
| 19. Somatic Symptoms |  |  |  |  |  |  |  |  |  |  |  |  |  |  |  |  |  |  | - | .29*** | .21* | .33*** | .26*** | .34*** | .26*** | .37*** | .09 |
| 20. Suicidal ideation |  |  |  |  |  |  |  |  |  |  |  |  |  |  |  |  |  |  |  | - | .31*** | .22** | .25*** | .41*** | .27*** | .45*** | .18 |
| 21. Psychosis |  |  |  |  |  |  |  |  |  |  |  |  |  |  |  |  |  |  |  |  | - | .19* | .22** | .33*** | .33*** | .29*** | .24** |
| 22. Sleep problems |  |  |  |  |  |  |  |  |  |  |  |  |  |  |  |  |  |  |  |  |  | - | .24** | .25*** | .16 | .25*** | .16 |
| 23. Memory |  |  |  |  |  |  |  |  |  |  |  |  |  |  |  |  |  |  |  |  |  |  | - | .33*** | .31*** | .36*** | .06 |
| 24.Repetitive Thoughts and Behaviors  25. Dissociation |  |  |  |  |  |  |  |  |  |  |  |  |  |  |  |  |  |  |  |  |  |  |  | - | .46***  - | .49***  .36*** | .13  .00 |
| 26. Personality Functioning  27. Substance Use |  |  |  |  |  |  |  |  |  |  |  |  |  |  |  |  |  |  |  |  |  |  |  |  |  | - | .08  - |

**p* < .05, ***p* < .01
